# MALP-2 pre-treatment modulates systemic inflammation in hemorrhagic shock

**DOI:** 10.1186/1476-9255-10-17

**Published:** 2013-04-12

**Authors:** Roman Pfeifer, Thomas Tschernig, Philipp Lichte, Derek Dombroski, Philipp Kobbe, Hans-Christoph Pape

**Affiliations:** 1 Department of Orthopaedic Trauma Surgery, RWTH Aachen University, Pauwelsstrasse 30, 52074 Aachen, Germany; 2 Institute of Anatomy and Cell Biology, Saarland University Faculty of Medicine, Homburg/Saar, Germany; 3 Parkland Health and Hospital Systems Department of Orthopaedic Surgery, Dallas, TX, USA

**Keywords:** Hemorrhagic shock, MALP-2, Pre-treatment, Systemic inflammation, TLR-2 agonist

## Abstract

**Background:**

TLR-2 is expressed on the surface of leucocytes, lung and liver tissue and initiates the activation of immune response after interaction with components of the bacterial cell wall. In this experiment we investigated whether immunostimulation with TLR-2 agonists under conditions of sterile inflammation (hemorrhagic shock (HS)) may affect the immune response and remote organ inflammation.

**Methods:**

Male C57/BL6 mice were subjected to standardized pressure-controlled HS (MAP of 35 mmHg for 90 minutes). The TLR-2 agonist macrophage-activated lipopeptide-2 (MALP-2) was administered (i.p.) either 12 hours prior to the induction of HS (Group MALP PT) or after the hypotensive period (90 minutes) (Group MALP T). After six hours, plasma cytokine levels (IL-6, KC, IL-10, and MCP-1) and lung and liver MPO activity were assessed.

**Results:**

Pre-treatment with MALP-2 resulted in a significant attenuation of the systemic pro-inflammatory (IL-6) response (MALP PT: 0.83±0.2 ng/ml vs. MALP T: 1.7±0.09 ng/ml) (p<0.05). In comparison to the liver MPO activity, lung MPO levels in in group MALP PT did not show differences to levels measured in MALP T mice (1.200±200 ng/mg vs. 1.800±200 ng/mg).

**Conclusions:**

After initial inflammation, MALP-2 pre-treatment was associated with attenuated systemic immune response after sterile stimulus. The TLR-2 agonist appears to affect sterile inflammation pathways. The exact mechanisms should be studied further to better understand these affects.

## Background

Trauma and hemorrhagic shock (HS) stimulate a systemic release of endogenous molecules that are known to activate the innate immune system [1]. These inflammatory mediators interact with Toll-like receptors (TLR), initiate the expression of transcription factors (e.g. NFκB) and provoke local and systemic liberation of pro- and anti-inflammatory cytokines [1,2]. Toll-like receptor 2 (TLR-2) is expressed on the surface of immune cells (leukocytes) and in the lung and liver. It has been shown that bacterial cell wall components are able to stimulate the systemic immune response through the TLR-2 pathway [3]. The associated imbalance between the pro- and anti-inflammatory immune system may result in either self-destructive hyper-inflammation or immune paralysis and sepsis [4]. Both are associated with the development of multiple organ failure (MOF) and high mortality rates [5,6]. Therefore, numerous studies have aimed to identify protective mechanisms that modulate the immune response following trauma or sepsis [7,8].

Macrophage-activating Lipopeptide-2 (MALP-2) is a lipopeptide and was primarily isolated from the cell wall of *Mycoplasma fermentans* and was synthetized chemically [9]. It is known to activate macrophages and other immune cells (e.g. B-cells) via TLR-2 / TLR-6 heterodimers [10]. The initial contact of MALP-2 and the immune system initiates a pro-inflammatory immune response [11-13]. However, desensitization of signal cascades was also observed in studies [3]. Pre-treatment with MALP-2 in mice with sepsis and peritonitis was associated with an attenuated immune response and reduced mortality rates [3]. Moreover, MALP-2 administration into the respiratory tract resulted in beneficial effects in murine pneumonia model with *Streptococcus pneumoniae*[14]. All these studies documented protective effects after MALP-2 treatment in infection models. The role of MALP-2 therapy in sterile inflammation has not been studied so far. To this end, we analyzed whether treatment with MALP-2 prior and after to an inflammatory stimulus attenuates the systemic inflammatory response and liver and lung inflammation in a murine hemorrhagic shock model.

## Methods

### Animals

Animals were housed in accordance with the regional animal research advisory committee guidelines and the experimental protocol has been approved by the Institutional Animal Use and Care Committee. Male C57/BL6 mice (Charles Rivers Laboratories, Germany) 6 to 10 weeks old and weighing 20 to 25 g were used for this study. Animals were maintained in the animal research center with a 12 hour light–dark cycle and had free access to laboratory feed and water.

### Murine hemorrhagic shock model

A standardized hemorrhagic shock model was performed as described previously [15,16]. Briefly, an incision on the left groin, dissection and cannulation of the femoral artery with sterile polyethylene tubing (PE-10) was performed. The tube was flushed with Heparin (Ratiopharm GmbH, Germany) to prevent clotting. Mean arterial pressure (MAP) was recorded using the digital blood pressure monitor (TSE Systems, Bad Homburg, Germany). Pressure controlled HS (MAP = 35 ± 5 mmHg, for 90 minutes) was performed by withdrawing blood over a 15-min interval via the arterial catheter. Animals were resuscitated over 15 minutes by transfusion of the removed blood and an equal volume of 0.9% saline. Thereafter the catheter was removed, the artery ligated and the skin incision closed. After a recovery phase of 4.5 hrs the animals were sacrificed.

### Group distribution

**Figure 1:**
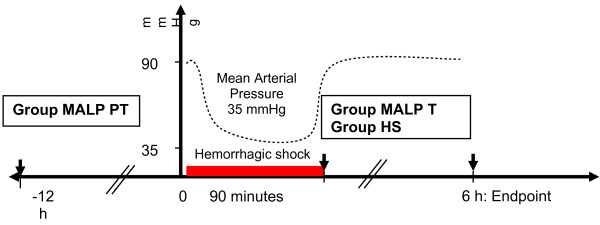
**Study design of experiment.** Animals were subjected into three treatment groups (n=6). *Group HS (Hemorrhagic Shock)* received 100 μl (intraperitoneal (i.p.) injection of phosphate buffered saline (PBS) 90 minutes after induction of HS, *Group MALP T (Treatment)* received an i.p. injection of MALP-2 (4 μg/kg BW) dissolved in 100 μl of PBS after the induction of HS, and Group MALP *PT (Pre-Treatment)* received an i.p. pre-treatment of MALP-2 twelve hours prior the initiation of HS. Mice were sacrificed 6 hours following HS.

Animals were randomly distributed to five experimental groups. Each group consisted of six mice and the endpoint in all experiments was six hours. *Control* animals were sacrificed directly after induction of anesthesia to obtain physiological baseline levels, and *sham* animals underwent a femoral artery catheterisation without drawing blood. Animals subjected to hemorrhagic shock were divided into three treatment groups (n=6). *Group HS* received 100 μl (intraperitoneal (i.p.) injection of phosphate buffered saline (PBS), *Group MALP T (Treatment)* received an i.p. injection of MALP-2 (4 μg/kg BW) dissolved in 100 μl of PBS after the induction of HS, and *Group MALP PT (Pre-Treatment)* received an i.p. pre-treatment of MALP-2 twelve hours prior the initiation of HS (Figure 1). MALP is synthetically synthesized and purchased from MALP Research, Braunschweig, Germany. MALP-2 was purified and diluted as described [17]. The stock solution was kept frozen under −20° and was thawed prior to administration.

### Plasma IL-6, IL-10, KC, and MCP-1

After six hours, thoracotomy (with anesthesia/ Pentobarbital and Isoflurane) was performed and mice were exsanguinated via cardiac puncture. At that time point, pronounced inflammatory response following hemorrhagic shock has been shown by prior studies [18]. Heparinized blood samples were centrifuged at 5,000 rpm for 10 min at 4°C. Thereafter, plasma was separated from cellular blood components and stored at −80°C until thawed for cytokine profile measurements. The systemic inflammation (Plasma Interleukin (IL)-6, IL-10, monocyte chemotactic protein 1 (MCP-1) and keratinocyte-derived chemokine (KC) levels) was evaluated using standardized ELISA kits (R&D System Inc., Mineapolis, MN, USA). Interleukin 6 and 10 are important prognostic parameters and both correlate with the systemic inflammatory response and injury severity [19-21]. MCP-1 was used due to recruitment and stimulation of monocytes, T-cells, and neutrophils [22]. Prior investigations have documented increased expression of MCP-1 during aseptic inflammation [23,24]. KC (IL-8 in humans) is a known marker of lung injury [25].

### Lung and liver myeloperoxidase activity (MPO)

Lung and liver tissue were immediately snap frozen at the experiment endpoint. Prior to the analysis, the tissues were thawed and homogenized in a lysis buffer as described by the manufacturer. MPO-enzyme-linked immunosorbend assay kits (MPO ELISA kit, Hycultec GmbH Beutelsbach, Germany) were used to quantify the myeloperoxidase (MPO) activity in lung and liver tissues. As only minor histological changes were observed following hemorrhagic shock models [15,16], we did not performed histological evaluation.

### Statistical analysis

Data were analysed using SPSS Version 18 (SPSS, Chicago, IL, USA). The null hypothesis was rejected for *P<* 0.05 (α =0.05). All results are expressed as the mean ± SE of six animals per group. Group comparisons were assessed using ANOVA (Bonferroni) in normally distributed variables.

## Results

### Plasma cytokine response

**Figure 2:**
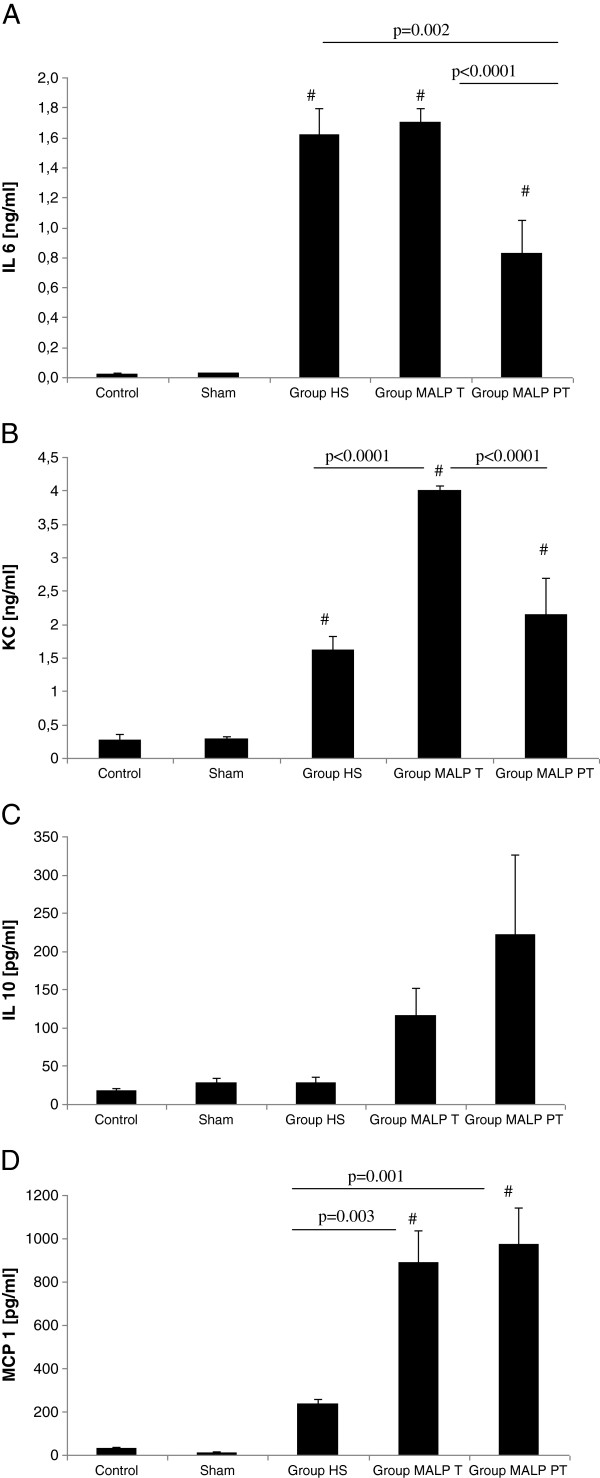
**Comparison of plasma IL-6 (A), KC (B), IL-10 (C), and MCP-1 (D) levels in C57/BL6 mice; Animals were subjected either to MALP-2 pre-treatment (12 hours) (*Group MALP PT*) or MALP-2 was administered following HS (90 minutes) (*Group MALP T*).***Group HS*: hemorrhagic shock with PBS administration after 90 minutes. Results are expressed as means ± SE of 6 animals per group (#<0.05 vs. Control/Sham).

The systemic IL-6 release was significantly elevated in all animals subjected to HS (Figure 2A). The i.p. injection of MALP-2 90 minutes following HS did not affect plasma IL-6 values when compared to untreated mice. In contrast, MALP-2 pre-treatment was associated with significantly lower IL-6 levels (p<0.0001). Six hours following HS, all animals demonstrated elevated systemic KC levels (Figure 2B). We measured a two-fold increase of plasma KC levels by i.p. administration of MALP-2 following HS (p<0.0001).

Mice treated with MALP-2 showed a slight increase of IL-10 (Figure 2C), but this difference was not statistically different. Regardless of the time MALP-2 was administered, comparable elevation of MCP-1 was observed in both groups (*Group MALP T* and *Group MALP PT*) (Figure 2D). The MCP-1 values were significantly lower in untreated mice as compared to levels measured in MALP-2 pre-treated mice and mice treated after HS.

### Organ inflammation

**Figure 3:**
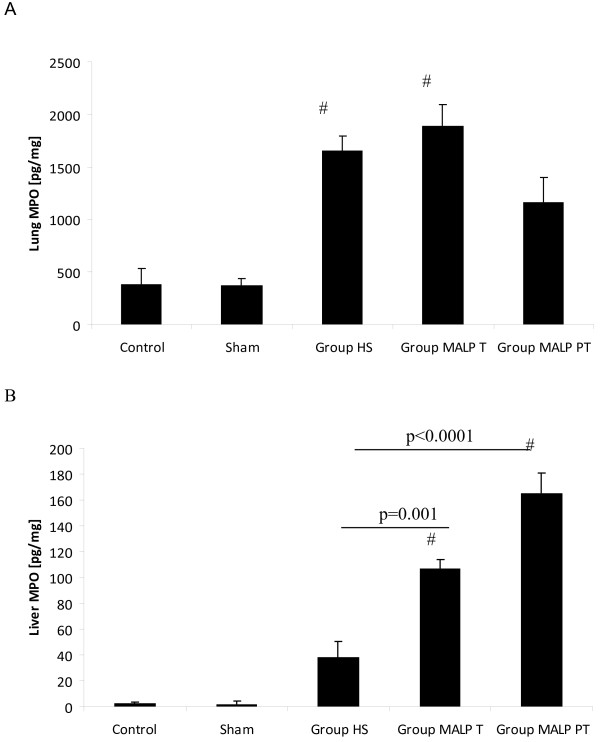
**Pulmonary (A) and liver (B) myeloperoxidase (MPO) activity in C57/BL6 mice; Animals were subjected either to MALP-2 pre-treatment (12 hours) (*Group MALP PT*) or MALP-2 was administered following HS (90 minutes) (*Group MALP T*).***Group HS*: hemorrhagic shock with PBS administration after 90 minutes. Results are expressed as means ± SE of 6 animals per group (#<0.05 vs. Control/Sham).

Elevated pulmonary MPO activity was measured in mice subjected to untreated HS and animals with MALP-2 treatment after HS (*Group MALP T*) (Figure 3A). Both groups have demonstrated comparable MPO activity levels. In pre-treated animals (*Group MALP PT*) MPO activity was decreased; however, not statistically significant as compared to either group HS and group MALP T. Compared to control and sham, lung MPO was increased by trend. The MPO activity in the liver showed a different pattern (Figure 3B). The highest liver MPO levels were measured in mice with MALP-2 pre-treatment (*Group MALP PT*). Untreated animals have demonstrated significantly lowed MPO activity in the liver when compared with MALP-2 treated study groups.

## Discussion

Numerous substances, including hormones (e.g. DHEA) [8], cytokines (e.g. IL-10) [26,27] and diets [28] have been identified to have immuno-modulatory effects. The administration of the gram-negative bacterial cell wall components prior to a second hit has been shown to diminish the severity of the inflammatory response [29]. This phenomenon was termed endotoxin tolerance. Similar to lipopolysaccharide (LPS), the injection of TLR-2 ligand (MALP-2) was associated with a decrease of pro-inflammatory mediators and immunomodulation [3]. It has been postulated that MALP-2 attenuates the immune response with a sufficient response for protective pathogen defense [3]. The consequences of MALP-2 treatment on the immune response were only studied in animals with bacterial infection [3,14,30]. The aim of these experiments was to analyze the role of MALP-2 treatment on sterile inflammation in response to HS.

This study revealed the following results: 1) Despite single and lowest administration dose the injection of MALP-2 prior to the induction of HS is feasible to attenuate the systemic IL-6 response. 2) Organ specific inflammation has been observed after the pre-exposure to MALP-2. While MPO activity within the lung tissue was tendentially decreased in pre-treated animals, the highest hepatic MPO levels were measured in the same group.

First, in contrast to KC values, the systemic release of IL-6 did not increase after i.p. injection of TLR-2 agonists (*Group MALP T*) when compared with levels in non-treated animals with HS (*Group HS*). This observation is in line with previous reports [3]. Feterowski et al. reported a slight increase in TNF-α levels and significant elevation of KC within 2 hours after MALP-2 injection [3]. Moreover, Reppe et al. reported comparable cytokine patterns within the lung tissue following inhalative MALP-2 administration [14]. Pre-exposure (12 hours) to MALP-2 prior to the HS (*Group MALP PT*) was associated with a significant reduction of plasma IL-6 levels in our study. Protective effects on the immune response after MALP-2 treatment have been already demonstrated in a murine sepsis model [30,31]. The administration of MALP-2 did not affect the levels of anti-inflammatory IL-10 in our study. These results are in line with finding from Feterowski et al., which described no elevation of IL-10 after systemic injection of MALP-2 in mice [3]. In addition, i.p. MALP-2 injection was associated with significantly elevated MCP-1 levels when compared to levels in mice without MALP-2 treatment. Our results are in line with findings reported by Kaufmann et al. [32]. Increased MCP-1 production accompanied by TNF-α was reported in human monocytes after MALP-2 administration. Moreover, our results indicate that the TLR-2 agonist appears to affect sterile inflammation pathways. TLR’s recognize gram-positive and gram-negative microbial components [33], which have been termed Pathogen-Associated Molecular Patterns (PAMPs), and generate a complex immune response [33]. In addition, endogenous tissue ligands (Damage-Associated Molecular Patterns (DAMPs)) were also identified and are known to induce systemic inflammation [1]. Authors have hypothesized that the immune system is mainly designed to identify threatening signals of either infection or injury, rather than differentiate between self and non-self [34]. However, it is still controversial whether the acute inflammatory response is activated by PAMPs or DAMPs through a universal pathway system. A similar pattern of acute inflammation after infectious and non-infectious stimulus has been described [18]. Moreover, there is increasing evidence that DAMP-mediated inflammation is generated by the TLR mechanisms [35]. In contrast, other studies pointed out DAMP specific pathways of the TLR activation [1,36].

Second, we identified an organ specific inflammation following MALP-2 administration. Intraperitoneal stimulation with MALP-2 12 hours prior to the initiation of HS was associated with tendentially decreased pulmonary MPO activity. This finding might be a consequence of the reduced systemic pro-inflammatory response recorded in our experiment. Moreover, this might be associated with changes in expression and sensitivity of TLRs within peritoneal cavity. Studies identified slight not significant increased TLR-4 expression within the spleen tissue after i.p. MALP-2 administration [3]. Moreover, TLR-2 up-regulation in the lung after inhalative MALP-2 application has been demonstrated [14]. The reduction of the pro-inflammatory mediators and pulmonary MPO activity might be accompanied by increased peritoneal inflammation and infiltration of neutrophils to a different compartment [14]. We observed a pronounced MPO activity within the liver in mice with MALP-2 treatment. MALP-2 is known to stimulate neutrophil recruitment to the site of application [10,37]. High liver MPO activity might be also related to increased influx of leukocytes into the peritoneal cavity and splanchnic circulation.

Treatment with TLR-2 agonists following HS does not attenuate the initial sterile inflammatory response in our study. In comparison to untreated mice, treatment with MALP-2 following HS was associated with increased levels of KC and MCP-1 and unchanged levels of IL-6. Moreover, the MPO activity could not be reduced. However, the therapeutic approach might be interesting in a presence of second hit (additional surgery) or infection (pneumonia/sepsis). Further studies are necessary to prove the therapeutic possibilities. In addition, in this study we focused on local and systemic inflammatory response. We did not determine the function or damage of organs such liver and kidney. Therefore, there is a lack of evidence of early multiple organ failure in this study.

Our analysis should be interpreted with respect to the following limitation: Only one time point and one treatment dosage have been assessed in our study. Therefore, no conclusions on dynamics of inflammation can be drawn out of these data. MALP-2 is dissolved in 30% 2-propanol / water for stock solution. However, we did not include 2-propanol to PBS in our control groups. It has to be considered that 2-propanol may influence the systemic inflammatory response.

## Conclusions

In this study, we showed that pre-treatment with TLR-2 agonists (MALP-2) may have beneficial effects in a model of HS that mimics sterile inflammation. These protective mechanisms were associated with reduced levels of pro-inflammatory cytokine (IL-6). Moreover, our results indicate that the TLR-2 agonist appears to affect sterile inflammation pathways. The exact mechanisms should be studied further. MALP-2 as a highly active biomolecule is not a “dirty drug” and can be synthetized under GMP conditions. It might be an option in strategies treating sterile inflammation after HS or severe injuries.

## Competing interests

This work has been supported by the Else Kröner-Fresenius Foundation. There are no further financial and non-financial competing interests.

## Authors’ contributions

All authors were involved in the research project and preparation of the manuscript. TT and HCP: They made a substantial contribution to the conception and design, and gave a critical and final approval. RP, PL, DD, and PK: They have performed the study, have collected the data and made an analysis and interpretation of these data. They also made a draft of the manuscript and revisions. All authors read and approved the final version of the manuscript.

## References

[B1] Piccinini AM Midwood KS DAMPening Inflammation by Modulating TLR Signalling Mediators Inflamm 2010 672395 21 10.1155/2010/672395 PMC2913853 20706656

[B2] Arslan F Keogh B McGuirk P Parker AE TLR2 and TLR4 in ischemia reperfusion injury Mediators Inflamm 2010 201 704202 20628516 10.1155/2010/704202 PMC2902053

[B3] Feterowski C Novotny A Kaiser-Moore GM Mühlradt PF Roßmann-Bloeck T Rump M Attenuated pathogenesis of polymicrobial peritonitis in mice after TLR2 agonist pre-treatment involves ST2 up-regulation Inter Immun 2005 17 1035 1046 10.1093/intimm/dxh282 16000329

[B4] Xiao W Mindrinos MN Seok J Cuschieri J Cuenca AG Gao H A genimic storm in critically injured humans J Exp Med 2011 208 2581 2590 10.1084/jem.20111354 22110166 PMC3244029

[B5] Baue AE Durham RM Faist E Systematic Inflammatory Response Syndrom (SIRS), Multiple Organ Dysfunction Syndrom (MODS), Multiple Organ Failure (MOF): Are we winning the Battle? Shock 1998 10 79 89 10.1097/00024382-199808000-00001 9721973

[B6] Ulvik A Kvale R Wentzel-Larsen T Flaatten H Multiple Organ Failure after Trauma affects even Long-Term Survival and Functional Status Crit Care 2007 11 1 8 10.1186/cc6111 PMC2556737 17784940

[B7] Neunaber C Zeckey C Andruszkow H Frink M Mommsen P Krettek C Immunomodulation in polytrauma and polymicrobial sepsis-where do we stand? Recent Pat Inflamm Allergy Drug Discov 2011 5 17 25 10.2174/187221311794474892 21158733

[B8] Angele MK Frantz MC Chaudry IH Gender and sex hormones influence the response to trauma and sepsis: potential therapeutic approaches Clinics 2006 61 479 488 17072448 10.1590/s1807-59322006000500017

[B9] Rharbaoui F Drabner B Borsutzky S Winckler U Morr M Ensoli B The Mycoplasma-derived lipopeptide MALP-2 is a potent mucosal adjuvant Eur J Immunol 2002 32 2857 2865 10.1002/1521-4141(2002010)32:10<2857::AID-IMMU2857>3.0.CO;2-R 12355438

[B10] Lührmann A Deiters U Skokowa J Hanke M Gessner JE Mühlradt PF In vivo effects of a synthetic 2-kilodalton macrophage-activating lipopeptide of Mycoplasma fermentans after pulmonary application Infect Immun 2002 70 3785 3792 10.1128/IAI.70.7.3785-3792.2002 12065522 PMC128036

[B11] Reppe K Tschernig T Luhrmann A van LV Grote K Zemlin MV Immunostimulation with macrophage-activating lipopeptide-2 increased survival in murine pneumonia Am J Respir Cell Mol Biol 2009 40 474 481 10.1165/rcmb.2008-0071OC 18931326

[B12] Pabst R Durak D Roos A Luhrmann A Tschernig T TLR2/6 stimulation of the rat lung: effects on lymphocyte subsets, natural killer cells and dendritic cells in different parts of the air-conducting compartments and at different ages Immunology 2009 126 132 139 10.1111/j.1365-2567.2008.02886.x 18565128 PMC2632703

[B13] Barrenschee M Lex D Uhlig S Effects of the TLR2 agonists MALP-2 and Pam3Cys in isolated mouse lungs PLoS One 2010 5 e13889 10.1371/journal.pone.0013889 21124967 PMC2987752

[B14] Reppe K Tschernig T Lührmann A van Laak V Grote K Zemlin MV Immunostimulation with macrophage-activating lipopeptide-2 increased survival in murine pneumonia Am J Respir Cell Mol Biol 2009 40 474 481 10.1165/rcmb.2008-0071OC 18931326

[B15] Pfeifer R Lichte P Schreiber H Sellei RM Schmidt J Dombroski D Inhalative vs. systemic IL-10 administration: Differences in the systemic inflammatory response and end-organ inflammation following hemorrhagic shock Cytokine 2012 60 266 270 10.1016/j.cyto.2012.05.028 22727902

[B16] Kobbe P Lichte P Schreiber H Reiss LK Uhlig S Pape HC Inhalative IL-10 attenuates pulmonary inflammation following hemorrhagic shock without major alterations of the systemic inflammatory response Mediators Inflamm 2012 201 2 512974 22046081 10.1155/2012/512974 PMC3199193

[B17] Mühlradt PF Kiess M Meyer H Süssmuth R Jung G Isolation, structure elucidation, and synthesis of a macrophage stimulatory lipopeptide from Mycoplasma Fermentans acting at picomolar concentration J Exp Med 1997 185 1951 1858 10.1084/jem.185.11.1951 9166424 PMC2196331

[B18] Chow CC Clermont G Kumar R Lagoa C Tawadrous Z Gallo D The acute inflammatory response indeverse shock states Shock 2005 24 74 84 10.1097/01.shk.0000168526.97716.f3 15988324

[B19] Gebhard F Pfetsch H Steinbach G Stecker W Kinzl L Brückner UB Is interleukin 6 an early marker of injury severity following major trauma in humans Arch Surg 2000 135 291 295 10.1001/archsurg.135.3.291 10722030

[B20] Akkose S Ozgurer A Bulut M Koksal O Ozdemir F Ozguc H Relationships between markers of inflammation, severity of injury, and clinical outcomes in hemorrhagic shock Adv Ther 2007 24 955 962 10.1007/BF02877699 18029320

[B21] Neidhardt R Keel M Steckholzer U Safret A Ungethuem U Trenz O Relationship of interleukin-10 plasma levels to severity of injury and clinical outcome in injured patients J Trauma 1997 42 863 871 10.1097/00005373-199705000-00017 9191668

[B22] van Zoelen MAD Verstege MI Draing C de Beer R van't Veer C Florguin S Endogenous MCP-1 promotes lung inflammation induced by LPS and LTA Mol Immunol 2011 48 1468 1476 10.1016/j.molimm.2011.04.001 21529952

[B23] Rosseau S Selhorst J Wiechmann K Leissner K Maus U Mayer K Monocyte migration through the alveolar epithelial barrier: adhesion molecule mechanisms and impact of chemokines J Immunol 2000 164 427 435 10605039 10.4049/jimmunol.164.1.427

[B24] Sandhir R Gregory E He Y Berman NE Upregulatation of inflammatory mediators in a model of chronic pain after spinal cord injury Neurochem Res 2011 36 856 862 10.1007/s11064-011-0414-5 21287269 PMC3189697

[B25] Hildebrand F Pape HC Krettek C The importance of cytokines in the posttraumatic inflammatory reaction Unfallchirurg 2005 108 793 803 10.1007/s00113-005-1005-1 16175346

[B26] Kobbe P Schmidt J Stoffels B Chanthaphavong RS Bauer AJ Pape HC IL-10 administration attenuates pulmonary neutrophil infiltration and alters pulmonary iNOS activation following hemorrhagic shock Inflamm Res 2009 58 170 174 10.1007/s00011-009-8116-z 19184345

[B27] Kobbe P Lichte P Schreiber H Reiss LK Uhlig S Pape HC Inhalative IL-10 Attenautes Pulmonary Inflammation following Hemorrhagic Shock without Major Alterations of the Systemic Inflammatory Response 2011 Mediators Inflamm Ref Type: In Press 10.1155/2012/512974 PMC3199193 22046081

[B28] Bastian L Weimann A Immunonutrition in patients after multiple trauma Br J Nutr 2002 87 S133 10.1079/BJN2001466 11895149

[B29] Morris M Li L Molecular mechanisms and pathological consequences of endotoxin tolerance and priming Arcch Immunol Ther Exp 2012 60 13 18 10.1007/s00005-011-0155-9 22143158

[B30] Zeckey C Tschernig T Hildebrand F Frink M Frömke C Dorsch M Macrophage-activating lipopeptide-2 exerts protective effects in a murine sepsis model Shock 2010 33 614 619 19940812 10.1097/SHK.0b013e3181cb8db4

[B31] Kerber-Momot T Leemhuis D Lührmann A Munder A Tümmler B Pabst R Beneficial effects of TLR-2/6 ligation in pulmonary bacterial infection and immunization with Pseudomonas aeruginosa Inflammation 2010 33 58 64 10.1007/s10753-009-9158-7 19844782

[B32] Kaufmann A Mühlradt PF Gemsa D Sprenger H Induction of cytokines and chemokines in human monocytes by Mycoplasma fermentans-derived lipoprotein MALP-2 Infect Immun 1999 67 6303 6308 10569741 10.1128/iai.67.12.6303-6308.1999 PMC97033

[B33] Arslan F Keogh B McGuirk P Parker A TLR2 and TLR4 in ischemia reperfusion injury 2010 Mediators Inflamm Ref Type: In Press 10.1155/2010/704202 PMC2902053 20628516

[B34] Matzinger P Tolerance, danger, and the extended family Annu Rev Immunol 1994 12 991 1945 10.1146/annurev.iy.12.040194.005015 8011301

[B35] Mollen KP Levy RM Prince JM Hoffmann RA Scott MJ Kaczorowski D Systemic inflammation and end organ damage following trauma involves functional TLR 4 signaling in both bone marrow-derived cells and parenchymal cells J Leukoc Biol 2008 83 80 88 17925504 10.1189/jlb.0407201

[B36] Liu Y Chen GY Zheng P CD24-Siglec G/10 discriminates danger-from pathogen-associated molecular patterns Trends Immunol 2009 30 557 561 10.1016/j.it.2009.09.006 19786366 PMC2788100

[B37] Deiters U Mühlradt PF Mycoplasmal lipopeptide MALP-2 induces the chemoattractant proteins macrophage inflammtory protein 1 alpha (MIP-1 alpha), monocyte chemoattractant protein 1 and MIP-2 and promotes leukocyte infiltration in mice Infect Immun 1999 67 3390 3398 10377117 10.1128/iai.67.7.3390-3398.1999 PMC116522

